# Unique Habitual Food Intakes in the Gut Microbiota Cluster Associated with Type 2 Diabetes Mellitus

**DOI:** 10.3390/nu13113816

**Published:** 2021-10-27

**Authors:** Yuriko Kondo, Yoshitaka Hashimoto, Masahide Hamaguchi, Shinto Ando, Ayumi Kaji, Ryosuke Sakai, Ryo Inoue, Saori Kashiwagi, Katsura Mizushima, Kazuhiko Uchiyama, Tomohisa Takagi, Yuji Naito, Michiaki Fukui

**Affiliations:** 1 Department of Endocrinology and Metabolism, Graduate School of Medical Science, Kyoto Prefectural University of Medicine, Kyoto 602-8566, Japan; yuri-k@koto.kpu-m.ac.jp (Y.K.); y-hashi@koto.kpu-m.ac.jp (Y.H.); shintoando@icloud.com (S.A.); kaji-a@koto.kpu-m.ac.jp (A.K.); sakaryo@koto.kpu-m.ac.jp (R.S.); michiaki@koto.kpu-m.ac.jp (M.F.); 2 Laboratory of Animal Science, Department of Applied Biological Sciences, Faculty of Agriculture, Setsunan University, Hirakata 573-0101, Japan; ryo.inoue@setsunan.ac.jp; 3 Molecular Gastroenterology and Hepatology, Graduate School of Medical Science, Kyoto Prefectural University of Medicine, Kyoto 602-8566, Japan; skashiwa@koto.kpu-m.ac.jp (S.K.); mizusima@koto.kpu-m.ac.jp (K.M.); k-uchi@koto.kpu-m.ac.jp (K.U.); takatomo@koto.kpu-m.ac.jp (T.T.); 4 Department for Medical Innovation and Translational Medical Science, Graduate School of Medical Science, Kyoto Prefectural University of Medicine, Kyoto 602-8566, Japan; 5 Department of Human Immunology and Nutrition Science, Kyoto Prefectural University of Medicine, Kyoto 602-8566, Japan; ynaito@koto.kpu-m.ac.jp

**Keywords:** dietary habits, fermented foods, gut microbiota, type 2 diabetes mellitus

## Abstract

This cross-sectional study aimed to clarify the characteristic gut microbiota of Japanese patients with type 2 diabetes (T2DM) using t-distributed stochastic neighbor embedding analysis and the k-means method and to clarify the relationship with background data, including dietary habits. The gut microbiota data of 383 patients with T2DM and 114 individuals without T2DM were classified into red, blue, green, and yellow groups. The proportions of patients with T2DM in the red, blue, green, and yellow groups was 86.8% (112/129), 69.8% (81/116), 76.3% (90/118), and 74.6% (100/134), respectively; the red group had the highest prevalence of T2DM. There were no intergroup differences in sex, age, or body mass index. The red group had higher percentages of the *Bifidobacterium* and *Lactobacillus* genera and lower percentages of the *Blautia* and *Phascolarctobacterium* genera. Higher proportions of patients with T2DM in the red group used α-glucosidase inhibitors and glinide medications and had a low intake of fermented soybean foods, including miso soup, than those in the other groups. The gut microbiota pattern of the red group may indicate characteristic changes in the gut microbiota associated with T2DM in Japan. These results also suggest that certain diabetes drugs and fermented foods may be involved in this change. Further studies are needed to confirm the relationships among traditional dietary habits, the gut microbiota, and T2DM in Japan.

## 1. Introduction

The number of patients with type 2 diabetes mellitus (T2DM) has been increasing worldwide, including in Japan. The main factors behind the global T2DM epidemic include overweight and obesity, a sedentary lifestyle, and an unhealthy diet [1].

Previous studies suggested that the gut microbiota affects the development of various diseases, such as T2DM, obesity, and inflammatory bowel disease [2,3,4,5]. The human gut microbiota is influenced by many factors, including diet, lifestyle, medications, and genetics [6]. Japanese individuals have a unique gut microbiota compared to other ethnic groups, which is characterized by a high proportion of the *Bifidobacterium* genera. The high proportion of the *Bifidobacterium* genera is considered to be the consequence of the intake of various saccharides in traditional and unique Japanese foods [7]. On the other hand, the typical diet in Japan is becoming increasingly Westernized [8,9]. Gut dysbiosis caused by changes in eating habits may be involved in the increased incidence of T2DM [10,11]. However, the association between T2DM and the gut microbiota and the relationship between lifestyle and changes in gut microbiota in Japanese populations have not been fully clarified. One reason for this is that gut microbiota data are vast and difficult to understand.

Recent developments in artificial intelligence technology have enabled the use of various machine-learning methods for analysis. One unsupervised machine learning method, t-distributed stochastic neighbor embedding (t-SNE), visualizes high-dimensional data via a nonlinear reduction to lower dimensions while retaining its original features [12]. Previous studies investigating the relationship between T2DM and the gut microbiota used methods such as principal component analysis, linear discriminant analysis effect size, and hierarchical clustering [2,6,13], but there have been no reports using t-SNE.

This study included a t-SNE analysis of the gut microbiota data of healthy Japanese individuals and patients with T2DM to create a gut microbiota panel. We then identified the groups associated with T2DM and examined their characteristics. We also investigated the relationship between each gut microbiota panel and the lifestyle factors in patients with T2DM.

## 2. Materials & Methods

### 2.1. Study Population and Data Collection

The Ethics Committee of Kyoto Prefectural University of Medicine (nos. ERB-C-534, RBMR-E-466-5, and ERB-C-1912) approved this study, which was conducted in accordance with the principles of the Declaration of Helsinki. Written informed consent was obtained from each participant prior to enrollment. None of the participants took antibiotics within 3 months prior to the study. A total of 522 individuals (114 without diabetes, 17 with type 1 diabetes, 383 with T2DM, and 8 with other types of diabetes) were enrolled between November 2016 and December 2017. Thus, the current study included 114 individuals without diabetes and 383 patients with T2DM.

Each participant’s height, body weight, and body mass index (BMI) were recorded. Patients with T2DM were then surveyed with respect to the medications used for diabetes, dyslipidemia, and hypertension, as well as proton pump inhibitor use. The diagnosis of T2DM was based on the Report of the Expert Committee on the Diagnosis and Classification of Diabetes Mellitus [14]. Information on maximum body weight, body weight at 20 years of age, family history of diabetes, and the duration of diabetes were obtained from the patients with T2DM. Based on the questionnaire responses, participants were categorized as non-, past, or current smokers. Regular exercisers were defined as those performing any kind of sport at least once a week [15].

Blood samples were collected from patients with T2DM for the analysis of hemoglobin A1c, fasting plasma glucose, creatine, and C-peptide levels. The glomerular filtration rate (GFR) was calculated using the following Japanese Society of Nephrology equation: estimated GFR (eGFR) = 194 × creatine^−1.094^ × age^−0.287^ (mL/min/1.73 m^2^) (×0.739 for women) [16]. Insulin resistance was evaluated by 20/(fasting C-peptide [ng/mL] × fasting plasma glucose [mg/dL]) [17]. The insulin secretion capacity was evaluated based on the secretory units of the islet cells in the transplantation index and the C-peptide immunoreactivity index [18]. Early morning spot urine samples were used to measure the urinary creatinine and albumin levels. The mean urinary albumin excretion was determined in three urine samples. Neuropathy was diagnosed according to the criteria of the Diagnostic Neuropathy Study Group [19]. Retinopathy was graded as follows: none, simple diabetic retinopathy, pre-proliferative diabetic retinopathy, or proliferative diabetic retinopathy [20].

Habitual dietary intake data were obtained from patients with T2DM using a brief self-administered diet history questionnaire [21,22]. Soybean food intake was summarized as tofu, fried tofu, and fermented soybean food, including natto and miso soup.

### 2.2. Sampling, DNA Extraction, Sequencing, and Data Analysis

Using previously described methods, the collection of fecal samples and analyses of gut bacterial composition were performed [23,24,25]. Briefly, collected fecal samples were preserved in a guanidine thiocyanate solution (Feces Collection kit; Techno Suruga Lab, Shizuoka, Japan). The isolation of genomic DNA was performed using a NucleoSpin Microbial DNA kit (Macherey-Nagel, Düren, Germany) according to the manufacturer’s instructions. Then, the purification of extracted DNA was performed using Agencourt AMPure XP beads (Beckman Coulter, Brea, CA, USA).

To generate sequencing libraries, a two-step polymerase chain reaction (PCR) of the purified DNA samples was performed. The first PCR was performed for amplification and used a 16S (V3–V4) Metagenomic Library Construction Kit for NGS (Takara Bio Inc., Kusatsu, Japan) with primer pairs 341F (5′-TCGTCGGCAGCGTCAGATGTGTATAAGAGACAGCCTACGGGNGGCWGCAG-3′) and 806 R (5′-GTCTCGTGGGCTCGGAGATGTGTATAAGAGACAGGGACTACHVGGGTWTCTAAT-3′) corresponding to the V3–V4 region of the 16S rRNA gene. The second PCR was performed to add the index sequences for the Illumina sequencer with a barcode sequence using the Nextera XT Index kit (Illumina, San Diego, CA, USA). The prepared libraries were sequenced for 250 paired-end sequences at Takara Bio’s Biomedical Center using the MiSeq Reagent v3 kit and MiSeq (Illumina) [25].

The generation of a table of the amplicon sequence variants (ASVs), including quality filtering and chimeric variant filtering, was performed using the DADA2 plugin of Quantitative Insights into Microbial Ecology 2 version 2019.4 [26]. Denoising by DADA2 was performed with the trimming length from the left set at 17 and from the right at 19. The truncation length was set to 250 for both reads. The taxonomy of each ASV was assigned using the sklearn classifier algorithm against the Greengenes database version 13_8. The singleton and ASVs assigned to the chloroplasts and mitochondria were removed in this study. The generation of a phylogenetic tree was performed using SATé-enabled phylogenetic placement [27]. Overall, 6,902 ASVs were obtained. The prediction of the functional profiles from the 16S rRNA dataset was performed using Phylogenetic Investigation of Communities by Reconstruction of Unobserved States version 2.1.4 [23] as previously described [25].

### 2.3. Strategy for Clustering Gut Microbiota

The ASVs were reduced to two dimensions using t-SNE in Python 3.7. Perplexity was determined using the perplexity-decidion.py command. The empirical value of perplexity is 5–50, and a perplexity of 10 was used in this analysis [28]. Two-dimensional ASVs were visualized as scatterplots and clustered using the k-means method. Based on the sum of the squared errors and the number of clusters, the optimal number of clusters was set to K = 4 using the elbow method. Four groups of two-dimensional ASVs were colored and visualized as red, blue, green, and yellow on the scatterplots and defined as the red, blue, green, and yellow groups, respectively.

### 2.4. Statistical Analysis

After clustering the gut microbiota into four groups, we compared the proportions of phyla and genera among them using the Kruskal–Wallis and Steel–Dwass tests. Furthermore, we compared age and BMI among the four groups using the Kruskal–Wallis test and the proportion of patients with T2DM and men among the four groups using the chi-square test. In addition, logistic regression analysis was performed to calculate the odds ratio for the prevalence of T2DM. Using only the data of patients with T2DM among all the participants, we evaluated the background, examination, and nutritional intake data of the four groups using the chi-square, Kruskal–Wallis, and Steel–Dwass tests. Statistical analyses were performed using JMP version 13.0 (SAS Institute Inc., Cary, NC, USA).

## 3. Results

**Figure 1:**
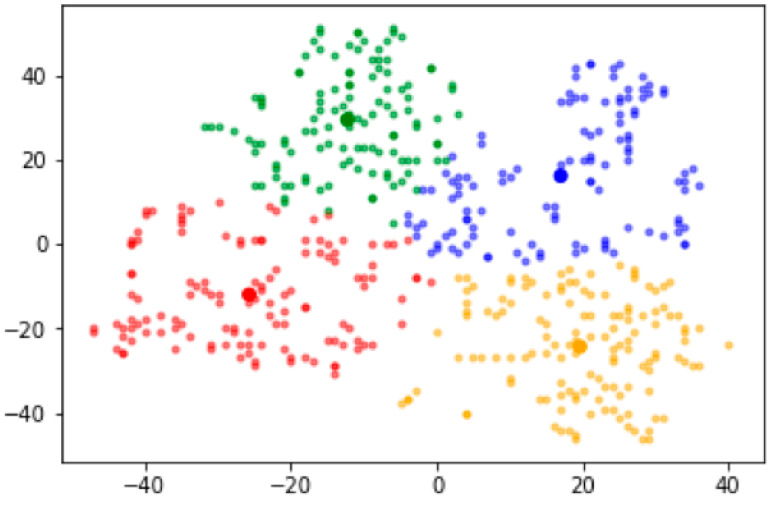
Gut microbiota panel according to the t-distributed stochastic neighbor embedding method.

This study analyzed the data of 497 individuals (114 without diabetes and 383 with T2DM). According to the t-SNE analysis, we divided the participants into four groups based on the gut microbiota sequencing data. Figure 1 shows a panel of two-dimensional ASVs divided into these four groups and colored red, blue, green, and yellow on the scatterplots. The proportions of patients with T2DM in the red, blue, green, and yellow groups in the t-SNE analysis were 86.8% (112/129), 69.8% (81/116), 76.3% (90/118), and 74.6% (100/134), respectively. Sex, age, and BMI did not differ among the groups (Table 1). A logistic regression analysis showed that the red group was associated with a higher prevalence of T2DM compared to the other groups even after adjusting for covariates (Table 2).

**Figure 2:**
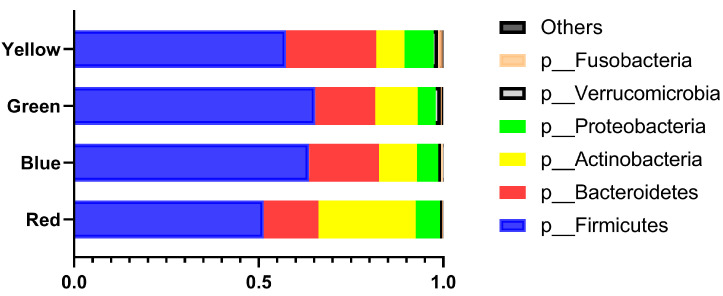
Phylum proportions by group. The differences among groups were evaluated using the Kruskal–Wallis and Steel–Dwass tests. Actinobacteria: red vs. the others, all *p* < 0.0001; blue vs. yellow, *p* = 0.0369; green vs. yellow, *p* < 0.0001. Bacteroidetes: red vs. blue, *p* = 0.0008; red vs. yellow, *p* < 0.0001; blue vs. green, *p* < 0.0001; blue vs. yellow, *p* < 0.0001; green vs. yellow, *p* < 0.0001. Firmicutes: red vs. blue and yellow, *p* < 0.0001; red vs. green, *p* = 0.0067; blue vs. yellow, *p* < 0.000; green vs. yellow, *p* < 0.0001.

Figure 2 shows the proportions of the phyla among the four groups. The proportion of the *Actinobacteria* phylum was higher in the red group than in the other groups, while the proportion of the *Firmicutes* phylum was lower in the red group than in the other groups.

**Figure 3:**
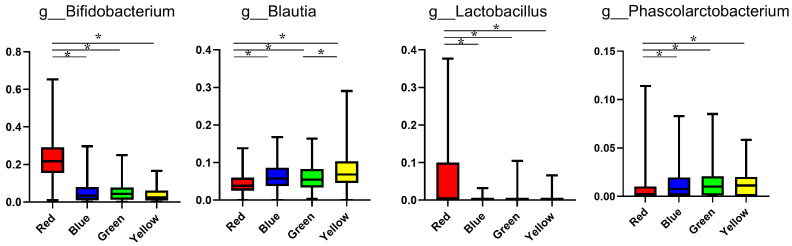
Gut microbiota at the genus level with significant differences seen in the red group. The proportions of genera among the four groups were evaluated using the Kruskal–Wallis and Steel–Dwass tests. * *p* < 0.05.

Figure 3 shows the differences in the proportions of genera among the four groups. The proportions of the *Bifidobacterium* and *Lactobacillus* genera were significantly higher in the red group than in the other groups, whereas the proportions of the *Blautia* and *Phascolarctobacterium* genera were significantly lower in the red group than in the other groups. The proportions of genera of all subjects are listed in Table S1.

Table 3 and Table 4 show the differences in the subjects’ characteristics among the four groups. The proportions of α-glucosidase inhibitor and glinide medication use were significantly higher in the red group than in the other groups.

Table 5 shows the differences in nutritional intake among the four groups. There were no intergroup differences in total energy intake. In contrast, the carbohydrate/energy intake (%) tended to be higher in the red group than in the other groups, although the difference was not statistically significant. In addition, the intake of fermented soybean foods, especially miso soup, was significantly lower, while the intakes of natto and Japanese rice wine, which are also fermented foods, tended to be lower in the red group than in the other groups.

## 4. Discussion

This study investigated the association between gut microbiota panels and T2DM and the relationship between gut microbiota panels and lifestyle factors. The gut microbiota panels were divided into four groups. Among them, the group with the highest prevalence of T2DM (red group) had a decreased proportion of the *Firmicutes* phylum and an increased proportion of the *Actinobacteria* phylum.

Moreover, patients in the gut microbiota group with the highest prevalence of T2DM reported a lower intake of fermented soybean foods, especially miso soup, and tended to have a lower intake of Japanese rice wine, a traditional Japanese fermented beverage. Moreover, a higher proportion of these patients were prescribed α-glucosidase inhibitors compared to those in the other groups.

Evidence collected over the past decade has shown the pivotal role of the gut microbiota in human health and diseases, including T2DM [2,3,4,5]. An association between the presence and/or proportion of bacteria and T2DM has been reported. For example, the abundance of the *Firmicutes* phylum was increased while that of the *Bacteroidetes* phylum was decreased in patients with T2DM [13,29]. However, there are vast data on the gut microbiota that are difficult to understand. In this study, we performed dimensionality reduction with t-SNE and divided the gut microbiota into four groups. The proportion of patients with T2DM was higher in the red group than in the other groups. The abundance of the *Firmicutes* phylum was significantly lower in the red group than in the other groups, which could be related to the increased abundance of the *Actinobacteria* phylum. The proportions of the *Bifidobacterium* and *Lactobacillus* genera were significantly higher in the red group than in the other groups. Previous studies demonstrated an association between these genera and T2DM [5,13,29]. A high proportion of the *Bifidobacterium* and *Lactobacillus* genera in Japanese patients with T2DM is associated with the use of α-glucosidase inhibitors [5]. Thus, α-glucosidase inhibitor use may be closely associated with T2DM-related gut microbiota.

A high-fat diet and low dietary fiber intake are associated with dysbiosis. The traditional diet in Japan is low in fat and high in fiber, characterized by the consumption of soybeans, vegetables, seaweed, fish, rice, and fermented foods. These factors may have formed the unique gut microbiota in the Japanese population. The gut microbiota of healthy Japanese individuals is specific, and the functional profiles of carbohydrate and energy metabolism also differ between Japanese individuals and those from other countries [7]. However, in Japan, the Westernization of food continues, and traditional food culture is being lost. Our recent study demonstrated that the gut microbiota and its functional profile differed between patients with T2DM and healthy individuals in Japan and that sucrose intake, which represents diet Westernization, affected gut dysbiosis in Japanese patients with T2DM [26]. In this study, fat and dietary fiber intake did not differ between patients in the gut microbiota group with the highest prevalence of T2DM (red group) and those in the other groups; however, the patients in the red group had a lower intake of fermented foods than those in the other groups. This result suggests that the decreased intake of traditional Japanese fermented foods caused by Westernization of the diet is related to gut dysbiosis in patients with T2DM. At the same time, the proportions of the *Blautia* genera were lower in the red group than in the other groups. Many Japanese fermented foods, such as fermented soybean paste, are prepared using the nonpathogenic fungus koji. Feeding mice glycosylceramide, which is abundant in koji, reportedly increased the proportion of the *Blautia* genera [30]. Thus, the lower percentage of the *Blautia* genera in the red group may be related to the lower intake of fermented foods.

This study has some limitations. A limited number of individuals without diabetes were included in the creation of the gut microbiota panel, and their dietary habits could not be evaluated. In addition, we did not sufficiently examine factors other than dietary content and diabetes medication as factors affecting the gut microbiota. Furthermore, because this was a cross-sectional study, the causal relationship between changes in the gut microbiota shown in this study and T2DM remains unknown. To clarify the relationship between dietary habits and gut dysbiosis in T2DM onset and progression, further studies of patients with T2DM who are not receiving antidiabetic medications and those with prediabetes are needed.

## 5. Conclusions

In this study, we visualized a huge amount of gut microbiota data by dimensionality reduction using t-SNE and divided them into four groups. We identified characteristic changes in the gut microbiota in patients with T2DM. Our findings suggested that certain diabetes drugs and fermented foods may be involved in these changes in the gut microbiota. To clarify the relationship between dietary habits and the gut microbiota, it will be necessary to reduce the influence of various medications.

## Figures and Tables

**Table 1:** Basic characteristics of all subjects.

Group	Red	Blue	Green	Yellow	*p* Value *
Number	129	116	118	134	-
Type 2 diabetes, % (*n*)	86.8 (112)	69.8 (81)	76.3 (90)	74.6 (100)	0.012
Male sex, % (*n*)	48.1 (62)	57.8 (67)	57.6 (68)	48.5 (65)	0.237
Age, years, mean (SD)	67.8 (10.9)	64.5 (12.5)	67.5 (10.6)	65.3 (11.2)	0.057
BMI, kg/m^2^, mean (SD)	23.8 (4.0)	24.2 (4.8)	23.3 (3.6)	23.6 (3.9)	0.424

BMI, body mass index; SD, standard deviation. * Kruskal–Wallis test was applied.

**Table 2:** Odds ratio for the prevalence of type 2 diabetes.

	Odds Ratio (95% CI)	*p* Value
Red group	Ref.	-
Blue group	0.34 (0.18–0.66)	0.001
Green group	0.46 (0.24–0.90)	0.024
Yellow group	0.47 (0.24–0.89)	0.021
Men	1.66 (1.08–2.55)	0.022
Age, years	1.01 (0.99–1.03)	0.257

**Table 3:** Characteristics of subjects with type 2 diabetes mellitus.

Group	Red	Blue	Green	Yellow	*p* Value *
Number	112	81	90	100	-
Male sex, % (*n*)	47.3 (53)	60.5 (49)	61.1 (55)	55.0 (55)	0.172
Age, years, mean (SD)	67.5 (10.9)	66.2 (10.9)	68.2 (9.5)	64.8 (10.9)	0.122
BMI, kg/m^2^, mean (SD)	24.1 (4.0)	25.0 (4.9)	23.8 (3.6)	24.2 (3.8)	0.295
Duration of diabetes, years, mean (SD)	14.8 (10.1)	13.6 (9.3)	15.1 (11.7)	12.2 (8.8)	0.164
Family history of diabetes, % (*n*)	46.4 (52)	40.7 (33)	37.8 (34)	53 (53)	0.16
Habitual alcohol intake, % (*n*)	5.4 (6)	13.6 (11)	5.6 (5)	18 (18)	0.006
Smoking status					0.403
Nonsmoker, % (*n*)	66.1 (74)	51.9 (42)	60 (54)	57 (57)	
Past smoker, % (*n*)	25 (28)	28.4 (23)	25.6 (23)	29 (29)	
Current smoker, % (*n*)	8.9 (10)	19.8 (16)	14.4 (13)	14 (14)	
Exercise, % (*n*)	42 (47)	50.6 (41)	44.4 (40)	54 (54)	0.293
Neuropathy, % (*n*)	24.1 (27)	28.4 (23)	24.4 (22)	21 (21)	0.721
Retinopathy, % (*n*)	23.2 (26)	22.2 (18)	27.8 (25)	12 (12)	0.052
Nephropathy, % (*n*)	43.8 (49)	49.4 (40)	44.4 (40)	35 (35)	0.252
History of cardiovascular disease, % (*n*)	18.8 (21)	12.4 (10)	18.9 (17)	6 (6)	0.025
Medication use					
Sulfonylurea, % (*n*)	25 (28)	16.1 (13)	24.4 (22)	24 (24)	0.452
Glinide, % (*n*)	16.1 (18)	7.4 (86)	3.3 (3)	4 (4)	0.002
Dipeptidyl peptidase-4 inhibitor, % (*n*)	59.8 (67)	43.2 (35)	54.4 (49)	42 (42)	0.029
Biguanide, % (*n*)	40.2 (45)	45.7 (37)	27.8 (25)	49 (49)	0.019
Thiazolidinedione, % (*n*)	6.3 (7)	4.9 (4)	3.3 (3)	0 (0)	0.096
α-Glucosidase inhibitor, % (*n*)	30.4 (34)	6.2 (5)	4.4 (4)	4 (4)	<0.001
Sodium glucose co-transporter 2 inhibitor, % (*n*)	14.3 (16)	23.5 (19)	15.6 (14)	13 (13)	0.238
Glucagon-like peptide-1 analog, % (*n*)	12.5 (14)	14.8 (12)	16.7 (15)	21 (21)	0.398
Insulin, % (*n*)	26.8 (30)	23.5 (19)	25.6 (23)	17 (17)	0.355
Renin angiotensin system inhibitor, % (*n*)	48.2 (54)	48.2 (39)	45.6 (41)	36 (36)	0.258
Calcium channel blocker, % (*n*)	32.1 (36)	25.9 (21)	31.1 (28)	23 (23)	0.427
Diuretic, % (*n*)	13.4 (15)	11.1 (9)	7.8 (7)	8 (8)	0.488
α blocker, % (*n*)	1.8 (2)	7.4 (6)	6.7 (6)	1 (1)	0.045
β blocker, % (*n*)	9.8 (11)	6.2 (5)	5.6 (5)	2 (2)	0.124
Statin, % (*n*)	42.0 (47)	40.7 (3)	36.7 (33)	35 (35)	0.71
Fibrate, % (*n*)	2.7 (3)	9.9 (8)	2.2 (2)	3 (3)	0.038
Eicosapentaenoic acid, % (*n*)	7.1 (8)	2.5 (2)	1.1 (1)	6 (6)	0.133
Ezetimibe, % (*n*)	2.7 (3)	7.4 (6)	2.2 (2)	0 (0)	0.282

BMI, body mass index; SD, standard deviation. * Kruskal–Wallis test was applied.

**Table 4:** Examination results of subjects with type 2 diabetes mellitus.

Group	Red	Blue	Green	Yellow	*p* Value *
Number	112	81	90	100	-
Systolic blood pressure, mmHg, mean (SD)	134.0 (17.7)	134.7 (19.3)	134.8 (20.0)	133.5 (18.3)	0.963
Diastolic blood pressure, mmHg, mean (SD)	77.9 (11.6)	80.3 (11.6)	78.9 (11.5)	78.7 (10.3)	0.524
Glucose, mg/dL (SD)	147.5 (50.9)	149.7 (46.5)	150.5 (50.0)	148.9 (49.0)	0.977
Hemoglobin A1c, % (SD)	7.30 (1.41)	7.54 (1.38)	7.38 (1.16)	7.27 (1.14)	0.508
C-peptide index, mean (SD)	1.23 (0.74)	1.33 (0.72)	1.18 (0.69)	1.26 (0.70)	0.628
Aspartate aminotransferase, IU/L, mean (SD)	23.8 (12.1)	26.1 (11.5)	21.2 (8.8)	23.7 (8.8)	0.026
Alanine aminotransferase, IU/L, mean (SD)	24.8 (19.6)	26.0 (16.4)	21.5 (15.1)	23.7 (14.1)	0.303
Gamma-glutamyltransferase, IU/L, mean (SD)	31.9 (24.3)	42.3 (48.4)	29.4 (20.8)	38.4 (29.0)	0.025
Creatinine, μmol/L (SD)	69.9 (30.8)	74.9 (25.7)	80.0 (44.1)	71.8 (23.2)	0.134
eGFR, mL/min/1.73 m^2^, mean (SD)	72.0 (21.4)	67.6 (16.4)	66.7 (20.8)	69.9 (17.7)	0.208
Uric acid, μmol/L, mean (SD)	299.7 (69.7)	306.1 (75.0)	306.1 (78.2)	317.7 (76.5)	0.373
Triglycerides, mmol/L, mean (SD)	1.44 (1.06)	1.63 (0.91)	1.37 (0.71)	1.60 (0.93)	0.16
HDL cholesterol, mmol/L, mean (SD)	1.62 (0.47)	1.53 (0.46)	1.50 (0.39)	1.56 (0.42)	0.278

eGFR, estimated glomerular filtration rate; HDL, high-density lipoprotein; SD, standard deviation. * Kruskal–Wallis test was applied.

**Table 5:** Nutritional intakes of subjects with type 2 diabetes mellitus.

Group	Red	Blue	Green	Yellow	*p* Value *
Number	112	81	90	100	-
Total energy intake, kcal/day, mean (SD)	1687 (621)	1804 (534)	1769 (733)	1714 (626)	0.294
Total energy intake, kcal/day/IBW, mean (SD)	30.5 (11.4)	31.6 (8.6)	29.6 (9.9)	29.4 (9.8)	0.29
Protein intake, g/day/IBW, mean (SD)	1.27 (0.58)	1.28 (0.41)	1.25 (0.47)	1.26 (0.52)	0.78
Protein intake/energy intake, % (SD)	16.6 (3.6)	16.3 (3.3)	17.1 (3.4)	17.1 (3.5)	0.341
Animal protein intake, g/day/IBW, mean (SD)	0.78 (0.47)	0.78 (0.32)	0.76 (0.36)	0.79 (0.41)	0.794
Vegetable protein intake, g/day/IBW, mean (SD)	0.50 (0.17)	0.50 (0.15)	0.49 (0.18)	0.47 (0.15)	0.402
Fat intake, g/day/IBW, mean (SD)	0.97 (0.49)	0.98 (0.32)	0.94 (0.37)	0.95 (0.36)	0.696
Fat intake/energy intake, % (SD)	28.6 (6.3)	28.0 (5.6)	28.8 (7.0)	29.0 (6.3)	0.811
Animal fat intake, g/day/IBW, mean (SD)	0.48 (0.31)	0.48 (0.19)	0.46 (0.21)	0.46 (0.23)	0.623
Vegetable fat intake, g/day/IBW, mean (SD)	0.49 (0.22)	0.50 (0.18)	0.48 (0.20)	0.49 (0.18)	0.925
Carbohydrate intake, g/day/IBW, mean (SD)	3.94 (1.41)	4.00 (1.27)	3.80 (1.49)	3.58 (1.37)	0.071
Carbohydrate intake/energy intake, % (SD)	52.2 (8.2)	50.7 (8.8)	51.2 (9.5)	48.8 (8.6)	0.06
Fiber intake, g/day, mean (SD)	11.8 (5.0)	11.9 (4.9)	13.0 (5.4)	12.1 (5.0)	0.337
Sucrose intake, g/day, mean (SD)	12.8 (8.6)	13.5 (9.7)	10.3 (8.6)	10.5 (6.7)	0.032
Salt intake, g/day, mean (SD)	10.5 (3.7)	10.9 (3.3)	10.9 (4.8)	10.7 (3.8)	0.778
Seaweed intake, g/day, mean (SD)	14.5 (17.0)	12.8 (12.4)	12.8 (13.0)	13.6 (13.7)	0.990
Soybean food intake, g/day, mean (SD)	160.5 (110.1)	174.0 (115.5)	202.7 (126.7)	186.5 (132.4)	0.048
Tofu and fried tofu intake, g/day, mean (SD)	51.2 (36.2)	48.4 (40.3)	52.1 (41.4)	47.7 (35.3)	0.689
Fermented soybean food intake, g/day, mean (SD)	109.3 (96.1)	125.6 (103.0)	150.7 (113.2)	138.9 (118.7)	0.011
Natto intake, g/day, mean (SD)	11.1 (15.7)	11.0 (14.3)	14.0 (16.7)	15.8 (20.3)	0.062
Miso soup intake, g/day, mean (SD)	98.2 (10.3)	114.6 (12.0)	136.7 (11.4)	123.0 (10.7)	0.03
Alcohol consumption, g/day, mean (SD)	4.75 (14.9)	11.1 (25.6)	5.23 (15.2)	10.71 (21.1)	0.011
Japanese sake intake, g/day, mean (SD)	4.08 (2.52)	9.38 (2.93)	4.79 (2.79)	3.21 (2.62)	0.06

IBW, ideal body weight; SD, standard deviation. * Kruskal–Wallis test was applied.

## Data Availability

The sequence data used in this study have been submitted to Sequence Read Archive (SRA) with the accession number PRJNA766337 (Available from 1 November 2021).

## Supplementary Materials

The following are available online at https://www.mdpi.com/article/10.3390/nu13113816/s1, Table S1: The proportions of genera of all subjects.
