# Supplementary material for: Unique Habitual Food Intakes in the Gut Microbiota Cluster Associated with Type 2 Diabetes Mellitus
Source: Nutrients. 2021 Oct 27;13(11):3816. doi: 10.3390/nu13113816 (PMC8621098; doi:10.3390/nu13113816)
Supplement: Supplementary file 1 [file nutrients-13-03816-s001.zip › nutrients-1440485-supplementary.pdf]

Supplemental Table:

Supplemental Table S1. The proportions of genera of all subjects.

Groups	Red	Blue	Green	Yellow
g__Bacteroides	0.107 (0.066)	0.093 (0.056)	0.061 (0.035)	0.221 (0.077)
g__Bifidobacterium	0.234 (0.123)	0.050 (0.051)	0.053 (0.048)	0.040 (0.039)
g__Blautia	0.045 (0.029)	0.066 (0.037)	0.059 (0.036)	0.079 (0.047)
g__Faecalibacterium	0.052 (0.044)	0.068 (0.046)	0.041 (0.034)	0.065 (0.063)
f__Lachnospiraceae;g__unknown	0.042 (0.031)	0.058 (0.032)	0.039 (0.026)	0.060 (0.044)
g__Ruminococcus	0.040 (0.051)	0.049 (0.052)	0.082 (0.053)	0.028 (0.031)
g__Collinsella	0.046 (0.042)	0.040 (0.037)	0.061 (0.041)	0.026 (0.027)
f__Ruminococcaceae;g__unknown	0.030 (0.030)	0.033 (0.026)	0.076 (0.056)	0.025 (0.034)
g__Roseburia	0.025 (0.033)	0.058 (0.060)	0.025 (0.031)	0.038 (0.041)
f__Lachnospiraceae;g__[Ruminococcus]	0.027 (0.021)	0.030 (0.022)	0.033 (0.028)	0.051 (0.044)
g__Streptococcus	0.030 (0.043)	0.045 (0.062)	0.018 (0.027)	0.026 (0.046)
g__Coprococcus	0.026 (0.023)	0.027 (0.021)	0.034 (0.027)	0.028 (0.026)
g__Oscillospira	0.020 (0.021)	0.020 (0.017)	0.061 (0.031)	0.017 (0.013)
g__Prevotella	0.011 (0.030)	0.076 (0.101)	0.019 (0.034)	0.007 (0.015)
g__Escherichia	0.026 (0.036)	0.013 (0.029)	0.017 (0.024)	0.041 (0.080)
g__Lactobacillus	0.056 (0.086)	0.004 (0.007)	0.004 (0.013)	0.003 (0.009)
g__Parabacteroides	0.013 (0.015)	0.015 (0.021)	0.017 (0.017)	0.018 (0.016)
g__Dorea	0.011 (0.020)	0.012 (0.019)	0.011 (0.011)	0.017 (0.020)
g__Megamonas	0.007 (0.024)	0.030 (0.071)	0.005 (0.017)	0.010 (0.027)
g__SMB53	0.008 (0.012)	0.011 (0.021)	0.022 (0.037)	0.009 (0.013)
g__Phascolarctobacterium	0.007 (0.014)	0.012 (0.014)	0.014 (0.015)	0.013 (0.012)
g__Akkermansia	0.005 (0.016)	0.009 (0.037)	0.019 (0.043)	0.007 (0.018)
o__Clostridiales;f__;g__	0.004 (0.009)	0.004 (0.008)	0.028 (0.037)	0.004 (0.011)
g__Citrobacter	0.007 (0.020)	0.016 (0.045)	0.008 (0.017)	0.009 (0.023)
g__Megasphaera	0.015 (0.034)	0.011 (0.023)	0.005 (0.012)	0.007 (0.016)

Data expressed as mean (SD).
